# Protective Effects of Atractylodis Rhizoma Extracts on Lung Injury Induced by Particulate Matter 2.5 in Mice

**DOI:** 10.3390/antiox14020127

**Published:** 2025-01-23

**Authors:** Eun-Hee Yun, Khawaja Muhammad Imran Bashir, Jeongjun Lee, Hunsuk Chung, Young-Sam Kwon, Jae-Suk Choi, Sae-Kwang Ku

**Affiliations:** 1 Department of Veterinary Surgery, College of Veterinary Medicine, Kyungpook National University, Daegu 41566, Republic of Korea; yehpic@knu.ac.kr (E.-H.Y.); kwon@knu.ac.kr (Y.-S.K.); 2 Department of Seafood Science and Technology, The Institute of Marine Industry, Gyeongsang National University, Tongyeong 53064, Republic of Korea; imranbashir@gnu.ac.kr; 3 German Engineering Research and Development Center for Life Science Technologies in Medicine and Environment, Busan 46742, Republic of Korea; 4 GAPI BIO Co., Ltd., Hwaseong 18622, Republic of Korea; orglab@gapibio.co.kr (J.L.); hunsukchung@dongbangchem.co.kr (H.C.); 5 Department of Anatomy and Histology, College of Korean Medicine, Daegu Haany University, Gyeongsan 38610, Republic of Korea

**Keywords:** antioxidant defense system, *Atractylodes japonica*, dexamethasone, natural respiratory refinement medicinal food ingredient, pulmonary protective effect

## Abstract

This study investigated the lung-protective effects of Atractylodis Rhizoma extracts (the root of *Atractylodes japonica* Koidz. ex Kitam), known as AJ extracts, in mitigating subacute pulmonary injuries caused by particulate matter 2.5 (PM_2.5_) exposure in Balb/c mice. AJ was given orally at concentrations of 400, 200, and 100 mg/kg, demonstrating a promising impact by mitigating oxidative stress and inflammation associated with phosphoinositide 3-kinase/protein kinase B (*PI3K/Akt*) and p38 mitogen-activated protein kinase α (*p38 MAPKα*) pathways and reducing mucus overproduction. These protective effects were achieved through the downregulation of *p38 MAPKα* and *PI3K/Akt* mRNA expressions, enhanced anti-inflammatory and antioxidant activities, and increased mucolytic expectorant effects arbitrated by elevated lung acetylcholine (ACh) and substance P levels, along with decreased mRNA expressions of *MUC5AC* and *MUC5B*. Importantly, these outcomes occurred without significant hepatotoxicity. While all AJ dosages provided dose-dependent pulmonary protection, their effects were less pronounced than those of dexamethasone (DEXA) at 0.75 mg/kg. However, AJ uniquely exhibited mucolytic expectorant activities absent in DEXA-treated mice. The results indicate that *A. japonica* may serve as a potential candidate for creating alternative treatments for respiratory conditions or as an ingredient in functional foods.

## 1. Introduction

The escalating levels of air pollution, particularly fine dust, in regions such as Korea, China, and Japan, pose a significant threat to public health and exacerbate climate change issues [1,2,3]. Beijing, China, is notably impacted, with substantial amounts of particulate matter originating from spring dust storms in areas like the Loess Plateau and Mongolian deserts, compounded by industrial emissions, vehicular traffic, and coal burning [4,5]. The particulate matter in Beijing predominantly consists of particulate matter 2.5 (PM_2.5_), which includes mineral dust, organic pollutants, and inorganic substances [6].

PM_2.5_, defined by its aerodynamic diameter of less than 2.5 µm, is a critical air pollutant linked to adverse health effects, including respiratory disorders, cardiovascular diseases, and lung cancer [7,8,9,10,11]. Its small size allows it to penetrate deep into the respiratory tract, reaching the alveoli, and the harmful chemicals it carries, such as endotoxins and heavy metals, further exacerbate its toxicity [12]. Consequently, there is an urgent need for innovative preventive and therapeutic measures to protect human respiratory health from PM-induced damage.

Medicinal herbs are gaining attention for their potential in developing new bioactive compounds [13,14]. *Atractylodes japonica* Koidz. ex Kitam, known as Sapju in Korean, is a traditional medicinal plant used for its health-promoting properties, including anti-inflammatory and antioxidant effects [15,16,17]. Preliminary studies suggest that extracts from Atractylodis Rhizoma (the root of *A. japonica* Koidz. ex Kitam) offer protective effects against PM_2.5_-induced lung injury in mice, attributed to their expectorant, anti-inflammatory, and antioxidant properties [18].

This study explores the effects of varying doses of Atractylodis Rhizoma extracts (AJ extracts) on lung injuries in mice caused by PM_2.5_, serving as a model to simulate human respiratory issues triggered by fine particulate matter [1,2,3]. Comparisons were made with dexamethasone (DEXA), a well-known anti-inflammatory drug, to assess AJ’s potential as an alternative therapeutic agent for respiratory health or as a functional food ingredient. The study aims to contribute to the development of effective alternative treatments for respiratory conditions exacerbated by air pollution [19,20,21].

## 2. Materials and Methods

### 2.1. Animal Care

This study involved eighty-eight healthy male SPF/VAF Inbred Balb/cAnNCrlOri (Balb/c) mice, six weeks old, sourced from OrientBio in Seungnam, Republic of Korea. The mice underwent a seven-day acclimation period before experiments commenced. They were housed in groups of four within polycarbonate cages under controlled conditions, maintaining a temperature of 20 °C to 25 °C and humidity at 30% to 35%, with a 12-h light cycle. Standard food (Cat. No. 38057; Purinafeed, Seungnam, Republic of Korea) and water were provided ad libitum. The mice were divided into six groups of ten, with 50 subjected to PM_2.5_-induced pulmonary injury and 10 serving as intact controls. Group selection was based on body weight measured one day before the first PM_2.5_ intranasal instillation, with intact mice averaging 20.80 ± 0.79 g and PM_2.5_-treated mice averaging 20.83 ± 1.23 g. The study adhered to international animal care standards and was approved by the Institutional Animal Care and Use Committee at Daegu Haany University [Approval No.: DHU2022-015, granted on 22 February 2022]. The experimental groups were organized as follows:

1. Intact (vehicle) control: Mice received 10 mL/kg of distilled water orally and 0.1 mL/kg of saline intranasally.
2. PM_2.5_ (vehicle) control: Mice received 10 mL/kg of distilled water orally and 1 mg/kg of PM_2.5_ intranasally.
3. DEXA: Mice received 0.75 mg/kg of DEXA (equivalent to 11.40 mg/kg DEXA-water soluble) orally and 1 mg/kg of PM_2.5_ intranasally.
4. AJ_400_: Mice received 400 mg/kg of AJ orally and 1 mg/kg of PM_2.5_ intranasally.
5. AJ_200_: Mice received 200 mg/kg of AJ orally and 1 mg/kg of PM_2.5_ intranasally.
6. AJ_100_: Mice received 100 mg/kg of AJ orally and 1 mg/kg of PM_2.5_ intranasally.

### 2.2. Induction of Lung Injuries in Mice Using Particulate Matter 2.5

Mice were subjected to subacute lung injuries by intranasal administration of PM_2.5_ suspensions, prepared at a concentration of 10 mg/mL in physiological saline. This procedure was performed using micropipettes with yellow tips. Instillations occurred twice, on day 0 and day 2, with a 48-h interval, each conducted one hour before the oral administration of test substances. The administered volume was 0.1 mL/kg, corresponding to a dose of 1 mg/kg [1,2,3]. To ensure uniform distribution of PM_2.5_ particles, the suspensions were sonicated for 30 min using an ultrasonicator (Model 5210; Branson, St. Louis, MO, USA) before each instillation. For the intact vehicle control group, mice received intranasal instillations of physiological saline at the same volume of 0.1 mL/kg, instead of PM_2.5_ suspensions, on both day 0 and day 2. This was performed to replicate the procedural stress of intranasal administration without PM_2.5_ exposure, ensuring consistent handling and stress conditions across all experimental groups.

### 2.3. Preparation and Administration of Test Substances

The test substance, a powdered 30% fermented ethanol extract of Atractylodis Rhizoma (the root of *Atractylodes japonica* Koidz. ex Kitam), referred to as AJ extracts, was standardized and supplied by NUTRACORE, Suwon, Republic of Korea. A detailed extraction procedure has been illustrated in Figure S1. AJ specimens are stored in the herbarium at the Medical Research Center for Herbal Convergence on Liver Disease, Daegu Haany University, Gyeongsan, Republic of Korea. DEXA, a synthetic glucocorticoid obtained from Sigma-Aldrich (St. Louis, MO, USA), served as a reference drug. Known for its potent anti-inflammatory properties, DEXA is significantly more effective than natural hydrocortisone and is used in treating various respiratory disorders, including PM_2.5_-induced lung injuries [19,20,21].

The AJ extract was dissolved in distilled water to prepare solutions at concentrations of 40, 20, and 10 mg/mL, corresponding to doses of 400, 200, and 100 mg/kg, respectively. These solutions were administered orally via gastric gavage using a 1 mL syringe with a zonde, at a volume of 10 mL/kg once daily for ten days. DEXA was similarly prepared in distilled water at 0.075 mg/mL, equivalent to a 0.75 mg/kg dose, and administered in the same manner. Control groups, including intact and PM_2.5_ controls, received equivalent volumes of distilled water to account for handling stress from oral gavage. The DEXA dose of 0.75 mg/kg was selected based on previous in vivo studies on its anti-inflammatory effects [19,20,21]. The intermediate AJ dose of 200 mg/kg was selected based on the intended clinical dose of 1 g for an adult human weighing 60 kg, considering the body surface area differences between humans and mice (1/12). This was calculated as follows: 1000 mg/60 kg × 12 = about 200 mg/kg. The highest and lowest AJ doses, 400 mg/kg and 100 mg/kg, respectively, were determined using a common ratio of 2, informed by prior screening tests in mice with PM_2.5_-induced pulmonary injuries [18], and in collaboration with NUTRACORE.

### 2.4. Analysis of Test Substance Using High-Performance Liquid Chromatography

The concentration of atractylenolide III in *A. japonica* root extracts was determined using an Agilent 1260 Infinity II high-performance liquid chromatography (HPLC) system (Agilent Technologies, Inc., Santa Clara, CA, USA). This system is equipped with a UV–visible detector and utilizes a Unison US-C18 column (dimensions: 4.6 mm × 250 mm, particle size: 5 μm; PeakmanSP Co., Ltd., Hanam, Republic of Korea). Both the AJ extract and the atractylenolide III standard were prepared in methanol and filtered through a 0.45 μm filter disc. The column was maintained at a steady temperature of 25 °C during the analysis, with atractylenolide III detection performed at a wavelength of 224 nm. The mobile phase was composed of acetonitrile and 0.05% trifluoroacetic acid. Detailed analytical conditions for the HPLC are provided in Table S1. Calibration was carried out using standard atractylenolide III (BF-A2011) from Biofron Inc., Fullerton, CA, USA, as a reference. The sample injection volume was 10 μL, with a flow rate set at 1.0 mL/min. Quantification was achieved by comparing the peak area of the sample to that of the standard, enabling determination based on the relationship between peak area and concentration.

### 2.5. Monitoring Body Weight Changes

Changes in body weight were tracked daily, beginning one day prior to the first nasal instillation of PM_2.5_ and extending throughout the entire experimental period. Measurements were obtained using an automatic electronic balance (Model XB320M; Precisa Instrument, Dietikon, Switzerland). To account for individual differences, body weight gain was calculated from the day of the first test substance administration to 24 h after the final administration of the test substance, as determined by Equation (1).

Body weight gain (g) over 10 Days = weight at 24 h after the final administration − weight at the beginning of administration(1)

### 2.6. Assessment of Serum Aspartate Aminotransferase and Alanine Aminotransferase Levels

Twenty-four hours following the last (10th) oral administration of the test substance, approximately 1 mL of venous blood was drawn from the vena cava. This procedure was conducted under anesthesia with 2 to 3% isoflurane (Hana Pharm. Co., Hwasung, Republic of Korea) in a gas mixture of 70% N_2_O and 28.5% O_2_, utilizing a rodent inhalation anesthesia system (Surgivet, Waukesha, WI, USA) and a rodent ventilator (Model 687; Harvard Apparatus, Cambridge, UK). Blood samples were centrifuged at 12,500 rpm for 10 min at 4 °C in a clot-activated serum tube using a cryo-centrifuge (Labocene 1236 MGR; Gyrozen, Daejeon, Republic of Korea). The serum was subsequently stored in an ultra-deep freezer (MDF-1156; Sanyo, Tokyo, Japan) at −150 °C until analysis. Serum aspartate aminotransferase (AST) and alanine aminotransferase (ALT) levels were quantified in international units per liter (IU/L) using an automated blood analyzer (Dri-Chem NX500i; Fuji Medical System Co., Ltd., Tokyo, Japan).

### 2.7. Measurements of Lung Weights

One day after the last (10th) oral administration of the test substance, the lungs of individual mice were excised under inhalation anesthesia. The absolute wet weights of the lungs were measured in grams using an automatic electronic balance (Precisa Instrument). To account for variations in individual body weights, relative lung weights were calculated as a percentage of body weight at the time of sacrifice, using Equation (2).

Relative lung weight (% body weight) = [lung wet weight/body weight at sacrifice (24 h post-final administration)] × 100(2)

### 2.8. Lung Sampling and Gross Inspections

Following the weighing of individual lungs, two 3-0 nylon ligations (using 3-0 sterilized nylon thread; NB 324; AILEE, Busan, Republic of Korea) were applied to the left secondary bronchus and the right lower secondary bronchus. The right upper and middle lobes were reserved for bronchoalveolar lavage fluid (BALF) collection, whereas the right lower lobes were used for analyzing matrix metalloproteinases (MMPs), acetylcholine (ACh), substance P, antioxidant defense mechanisms, lipid peroxidation, reactive oxygen species (ROS), and cytokines. The lobes left were reserved for gross examination, histopathological evaluation, and Realtime RT-PCR analysis.

### 2.9. Bronchoalveolar Lavage Fluid Collection and Cytological Analysis

After applying 3-0 nylon ligatures, 1 mL of physiological saline was introduced into the tracheal cannula (20 G) and then withdrawn using a syringe. This process was repeated twice to collect two samples from each animal, following previously established methods with minor modifications [20,22]. Total cell counts were determined with an automated cell counter (Countess C10281; Invitrogen, Carlsbad, CA, USA) using trypan blue staining (Sigma-Aldrich, St. Louis, MO, USA). The 10-day experimental period represents a subacute phase in pathogenesis, prompting the inclusion of macrophages as a type of monocyte. Additionally, total leukocyte numbers and differential counts—including neutrophils, lymphocytes, monocytes, and eosinophils—were evaluated using an automated hematology cell counter (Cell-DYN3700; Abbott Laboratories, Abbott Park, IL, USA).

The right lower lung lobes were homogenized with a bead beater (TacoTMPre, Gene-Research Biotechnology Corp., Taichung, Taiwan) and an ultrasonic cell disruptor (KS-750; Madell Technology Corp., Ontario, CA, USA) in an equal volume of normal saline. The homogenates were kept at −150 °C in an ultra-deep freezer (MDF-1156; Sanyo, Tokyo, Japan) until analysis. Lung tissue homogenates were centrifuged at 12,500 rpm for 30 min at 4 °C using a cryocentrifuge (Gyrozen). The supernatants were analyzed for tumor necrosis factor (TNF-α; MBS843393), interleukin 6 (IL-6; MBS843429), chemokine (C-X-C motif) ligand 1 (CXCL-1; MBS824609), chemokine (C-X-C motif) ligand 2 (CXCL-2; MBS824972), matrix metalloproteinase-9 (MMP-9; MBS2512650), matrix metalloproteinase-12 (MMP-12; MBS2508678), substance P (MBS843429), and ACh (MBS284198) using enzyme-linked immunosorbent assay (ELISA) kits specific to each analyte (Mybiosource, San Diego, CA, USA) following the manufacturer’s instructions. Optical density was measured at 450 nm with a microplate reader (Sunrise, Tecan, Männedorf, Switzerland).

### 2.10. Measurement of Lung Lipid Peroxidation

Lung tissue homogenates were mixed with 0.01 M Tris-HCl buffer (pH 7.4) and centrifuged at 12,000× *g* for 15 min at 4 °C using a cryocentrifuge (Gyrozen), as reported by Kavutcu et al. [23]. The extent of lipid peroxidation in the lungs was assessed by measuring malondialdehyde (MDA) levels using the thiobarbituric acid reactive substances (TBARS) assay. Absorbance was checked at 525 nm with a UV/Vis spectrophotometer (OPTIZEN POP; Mecasys, Daejeon, Republic of Korea), and the values were expressed as nanomoles of MDA per milligram of protein [24]. Total protein content was determined using the Lowry method [25], with bovine serum albumin (Invitrogen, Carlsbad, CA, USA) as the standard.

### 2.11. Measurement of Lung Reactive Oxygen Species (ROS) Levels

ROS levels in lung tissue homogenates were evaluated using 2,7′-dichlorofluorescein diacetate (DCFDA) as a fluorescent probe. The analysis was performed with the Cellular Reactive Oxygen Species Detection Assay Kit (ab113851; Abcam, Cambridge, MN, USA), according to the manufacturer’s guidelines. Fluorescence intensity was measured at excitation and emission wavelengths of 490/520 nm using a fluorescence microplate reader (Versa-MaxTM; Molecular Devices, Sunnyvale, CA, USA). Fluorescence values were expressed in relative fluorescence units (RFUs) and normalized to protein concentrations, reported as RFU per microgram of protein [26].

### 2.12. Measurement of Lung Antioxidant Defense Systems

To evaluate the antioxidant defense mechanisms in the lungs, homogenates were treated with 0.1 mL of 25% trichloroacetic acid (Merck, West Point, CA, USA) and centrifuged at 4200 rpm for 40 min at 4 °C. Glutathione (GSH) levels were measured spectrophotometrically at 412 nm using 2-nitrobenzoic acid (Sigma-Aldrich, St. Louis, MO, USA), following the method described by Sedlak and Lindsay [27]. Catalase (CAT) activity was determined by observing the breakdown of hydrogen peroxide (H_2_O_2_) at 240 nm, where one unit of CAT activity is defined as the enzyme amount needed to decompose 1 nM of H_2_O_2_ per minute at 25 °C and pH 7.8 [28]. Superoxide dismutase (SOD) activity was evaluated by its ability to inhibit the formation of formazan dye from nitrotetrazolium blue by superoxide radicals produced from xanthine and xanthine oxidase. The level of inhibition was measured at 560 nm, with results expressed as units per milligram of protein. One unit of SOD activity corresponds to a 50% decrease in absorbance [29].

### 2.13. Real-Time RT-PCR

The mRNA expression levels of genes associated with mucus production (*MUC5B* and *MUC5AC*), inflammation (*p38 MAPKα*, *NF-κB1*, *Akt1*, *PI3K*, and *PTEN*), and cell apoptosis (*Bcl-2* and *Bax*) were quantified using Real-time RT-PCR, as reported in earlier studies [30,31]. RNA was extracted using Trizol reagent (Invitrogen, Carlsbad, CA, USA). The concentration and quality of RNA were evaluated using the Real-time System (CFX96TM; Bio-Rad, Hercules, CA, USA). To remove DNA contamination, samples were treated with the DNA-free DNA Removal Kit (Cat. No. AM1906; Thermo Fisher Scientific Inc., Rockford, IL, USA), and cDNA was synthesized using the High-Capacity cDNA Reverse Transcription Kit (Cat No. 4368813; Thermo Fisher Scientific Inc., Rockford, IL, USA), following the manufacturer’s instructions.

Quantitative PCR was performed with the ABI Step One Plus Sequence Detection System (Applied Biosystems, Foster City, CA, USA). The PCR cycling conditions were as follows: an initial pre-denaturation at 95 °C for 1 min, followed by 50 cycles of denaturation at 95 °C for 15 s, annealing at 55–65 °C for 20 s, and extension at 72 °C for 30 s. β-actin served as an internal control to verify sample integrity. The sequences of the PCR primers are listed in Table S1. For quantitative analysis, mRNA levels were normalized to the control lung tissue, and the relative expression of the target genes was calculated using the 2^−ΔΔCq^ method [32].

### 2.14. Histopathological Analysis

For histopathological examination, approximately equal regions from the left lateral lobe of each lung were trimmed. These sections were re-fixed in 10% neutral buffered formalin for a minimum of 24 h. After fixation, the tissues were processed into paraffin blocks using an automated tissue processor (Shandon Citadel 2000; Thermo Scientific, Waltham, MA, USA) and embedded with an embedding center (Shandon Histostar, Thermo Scientific, Waltham, MA, USA). Sections with a thickness of 3 to 4 μm were stained with hematoxylin and eosin (H&E) for general histopathological evaluation and with Periodic A Acid–Schiff (PAS) stain for identifying mucus-producing goblet cells, following well-reported protocols [20,33,34]. The histological profiles were examined under a light microscope (Model Eclipse 80i; Nikon, Tokyo, Japan). Observers were blinded to group distribution during analysis.

Detailed histomorphometric analyses included measurements of the mean Alveolar Surface Area (ASA) as an indicator of gas exchange capacity [20,22,35], the number of PAS-positive mucus-producing cells in the secondary bronchus, mean alveolar septal thickness (μm), and the number of inflammatory cells in alveolar regions (cells/mm^2^) [20,22,34,36]. Inflammatory cells attached to the alveolar surface or located on the alveolar space were counted as ×10 cells/mm^2^. Histological fields were selected from the upper regions of the secondary bronchus, with at least one field analyzed per left lung tissue, totaling at least 10 histological fields per group.

### 2.15. Statistical Analysis

Data were expressed as mean ± standard deviation (S.D.) for groups consisting of 10 mice each. To evaluate differences among various dosage groups, multiple comparison tests were employed. Levene’s test was utilized to check for variance homogeneity. If variance was homogeneous, as indicated by Levene’s test, a one-way analysis of variance (ANOVA) was conducted, followed by Tukey’s Honest Significant Difference (THSD) test to pinpoint significant group differences. In the event of variance inhomogeneity indicated by Levene’s test, Dunnett’s T3 (DT3) test was used for pairwise comparisons [37,38]. Statistical significance was determined at *p* < 0.05. Analyses were executed using SPSS version 18.0 (SPSS Inc., Chicago, IL, USA). Furthermore, percentage changes were calculated to evaluate the severity of lung damage induced by PM_2.5_ by comparing the intact vehicle and PM_2.5_ control groups. Efficacy was assessed by calculating percentage changes between the PM_2.5_ control group and test groups treated with AJ at 400, 200, and 100 mg/kg, or DEXA at 0.75 mg/kg, using Equations (3) and (4), as described in previous studies [20,39,40].

Percentage change relative to intact vehicle control (%) = [(value for PM_2.5_ control − value for intact vehicle control)/value for intact vehicle control] × 100(3)

Percentage change relative to PM_2.5_ control (%) = [(value for test substance or reference-treated group − values for PM_2.5_ control)/value for PM_2.5_ control] × 100 (4)

## 3. Results

### 3.1. Atractylenolide III Concentration in AJ Extract

**Figure 1:**
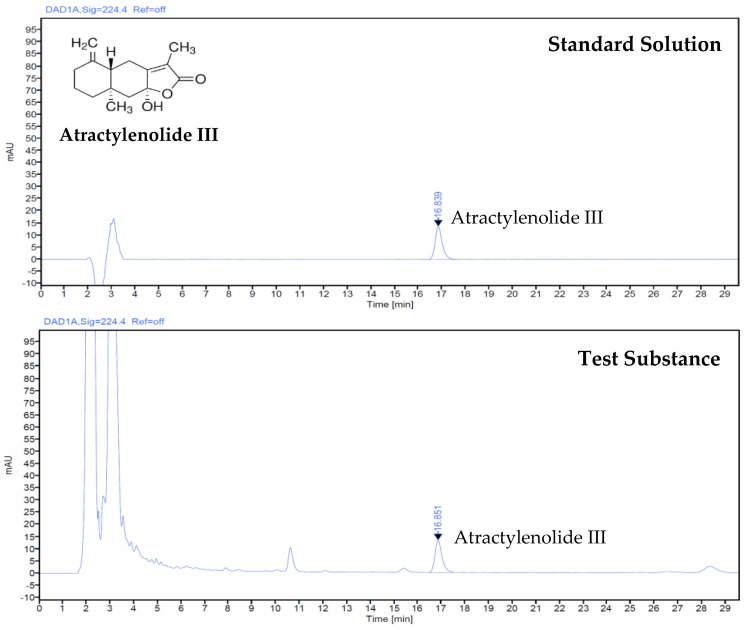
HPLC chromatogram displaying atractylenolide III in both standard solution and root extracts of *Atractylodes japonica* Koidz. ex Kitam.

Using HPLC analysis, the concentration of atractylenolide III in the AJ extract was quantified at 0.56 mg per gram. This measurement was accomplished by evaluating the relative peak area against the standard peak area and concentration, as depicted in Figure 1.

### 3.2. Changes in Body Weight and Weight Gain

**Figure 2:**
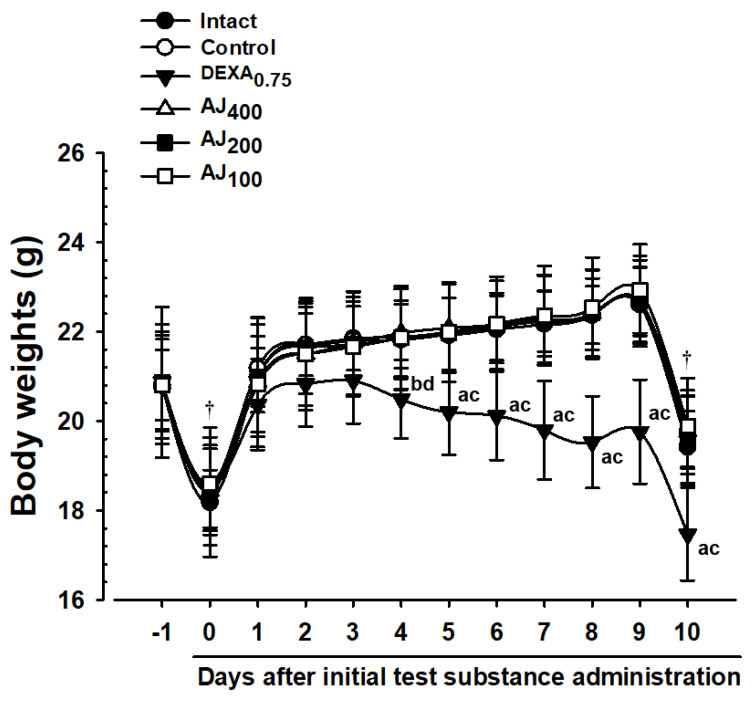
Body weight changes in mice with intact or PM_2.5_-induced pulmonary injury. Values are presented as means ± SD for 10 mice per group. PM_2.5_ refers to diesel particulate matter NIST 1650b; DEXA denotes dexamethasone; AJ stands for Atractylodis Rhizoma, the root extract of *Atractylodes japonica* Koidz. ex Kitam; THSD represents Tukey’s Honest Significant Difference. ‘Day-1’ indicates one day prior to the initial administration of the test substance, while ‘Day 10’ represents the day of sacrifice, 24 h after the final administration of the test substance. All animals were fasted overnight before the initial administration of the test substance and before sacrifice (†). Significance is marked as ^a^ *p* < 0.01 and ^b^ *p* < 0.05 when compared with the intact vehicle control using the THSD test; ^c^ *p* < 0.01 and ^d^ *p* < 0.05 when compared with the PM_2.5_ control using the THSD test.

During the 10-day experimental period, no significant changes in body weight or weight gain were observed among the mice instilled with PM_2.5_ intranasally compared to the intact vehicle control, except for the group receiving DEXA (0.75 mg/kg). In this DEXA-treated group, notable decreases in body weight were detected starting from the fourth day after the initial oral administration, reaching statistical significance. Consequently, the overall body weight gain over the 10 days was significantly lower than in the intact vehicle control group. In contrast, mice treated with the test substance AJ (400–100 mg/kg) showed no significant differences in body weight or weight gain throughout the period compared to the PM_2.5_ control mice. The significant reduction in body weight and weight gain observed in the DEXA group highlights its pronounced effect compared to other groups (Figure 2; Table S2).

### 3.3. Changes in Gross Observations and Lung Weights

**Figure 3:**
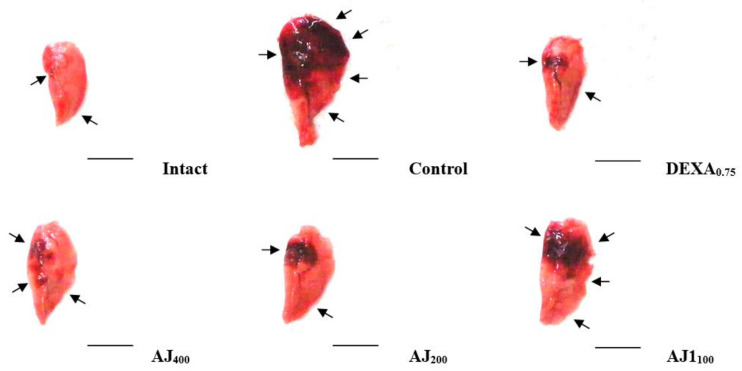
Representative images of the gross lung—left lobe from mice with intact or PM_2.5_-induced pulmonary injury. (**Intact**) mice orally administered with distilled water and given saline intranasal instillation, (**Control**) mice orally administered with distilled water and given PM_2.5_ intranasal instillation, (**DEXA_0.75_**) mice orally administered with 0.75 mg/kg DEXA and given PM_2.5_ intranasal instillation, (**AJ_400_**) mice orally administered with 400 mg/kg AJ and given PM_2.5_ intranasal instillation, (**AJ_200_**) mice orally administered with 200 mg/kg AJ and given PM_2.5_ intranasal instillation, (**AJ_100_**) mice orally administered with 100 mg/kg AJ and given PM_2.5_ intranasal instillation. PM_2.5_ refers to diesel particulate matter NIST 1650b; DEXA denotes dexamethasone; AJ stands for Atractylodis Rhizoma, the root extract of *Atractylodes japonica* Koidz. ex Kitam. Arrows point to regions of congestion. Scale bars represent 6.00 mm.

During gross examination, the PM_2.5_ control group exhibited notable focal lung congestion and enlargement compared to the intact vehicle control group, which corresponded with significant increases in both absolute and relative lung weights. The administration of AJ at all three doses significantly reduced these parameters, demonstrating a dose-dependent inhibitory effect on PM_2.5_-induced lung changes. While all AJ doses were effective, these effects were somewhat less pronounced than those observed with DEXA (0.75 mg/kg), which showed the strongest protective effect against lung congestion and weight increases (Figure 3; Table 1).

### 3.4. Bronchoalveolar Lavage Fluid (BALF) Cytology

In the PM_2.5_ control mice, there were significant increases (*p* < 0.01) in the numbers of total cells, lymphocytes, total leukocytes, monocytes, eosinophils, and neutrophils in the BALF compared to the intact vehicle control mice. However, the oral administration of AJ at all three doses significantly reduced (*p* < 0.01) these PM_2.5_-induced increases in BALF cellular components in a dose-dependent manner relative to the PM_2.5_ control group. All three doses of AJ demonstrated inhibitory effects on PM_2.5_-induced pulmonary injury-related BALF cytological changes, although these effects were somewhat less pronounced than those observed with DEXA (0.75 mg/kg) under the conditions of this study (Table 2).

### 3.5. Serum Serum Aspartate Aminotransferase and Alanine Aminotransferase Levels

**Figure 4:**
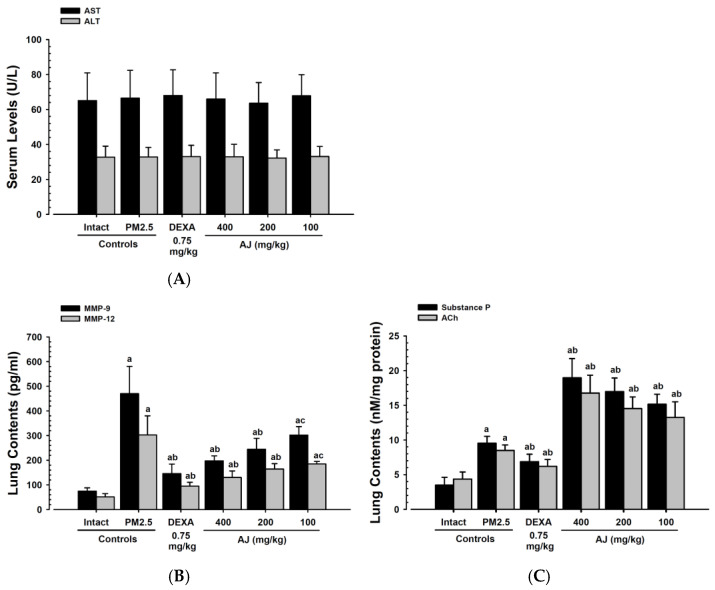
Serum AST and ALT (**A**) levels, lung MMP-9 and MMP-12 (**B**), and lung ACh and substance P levels (**C**) in mice with intact or PM_2.5_-induced pulmonary injury. Values are presented as means ± SD for groups of 10 mice. PM_2.5_ refers to diesel particulate matter NIST 1650b; DEXA denotes dexamethasone; AJ indicates Atractylodis Rhizoma, the root extract of *Atractylodes japonica* Koidz. ex Kitam; AST stands for aspartate aminotransferase; ALT represents alanine aminotransferase. MMP represents matrix metalloproteinase; ACh represents acetylcholine; DT3 is Dunnett’s T3. Statistical significance is indicated as ^a^ *p* < 0.01 when compared with the intact vehicle control using the DT3 test; ^b^ *p* < 0.01 and ^c^ *p* < 0.05 when compared with the PM_2.5_ control using the DT3 test.

There were no significant changes in AST and ALT levels in the PM_2.5_ control mice compared to the intact vehicle control mice. Similarly, no significant changes in serum AST and ALT levels were observed in mice treated with DEXA (0.75 mg/kg) or AJ at doses of 400, 200, and 100 mg/kg when compared to the PM_2.5_ control group under the conditions of this study (Figure 4A). The percentage change in serum AST levels for the PM_2.5_ control group was 2.15% relative to the intact vehicle control. In comparison, the changes for the DEXA (0.75 mg/kg) and AJ at 400, 200, and 100 mg/kg were 2.26%, −0.75%, −4.36%, and 1.95%, respectively, compared to the PM_2.5_ control group. The percentage change in serum ALT levels for the PM_2.5_ control group was 0.31% compared to the intact vehicle control. The changes for the DEXA (0.75 mg/kg) and AJ at 400, 200, and 100 mg/kg were 0.61%, 0.30%, −1.83%, and 0.91%, respectively, compared to the PM_2.5_ control group.

### 3.6. Lung Cytokine Levels: IL-6, TNF-α, CXCL1, and CXCL2

Significant increases in lung tissue cytokines IL-6, TNF-α, CXCL1, and CXCL2 were observed in PM_2.5_ control mice compared to intact vehicle control mice. The oral administration of AJ at all three doses significantly inhibited these PM_2.5_-induced cytokine elevations in a dose-dependent manner. Notably, AJ at 400 mg/kg reduced TNF-α by 60.59% and IL-6 by 66.59% compared to the PM_2.5_ control group. Although effective, these reductions were less pronounced than those achieved with DEXA (0.75 mg/kg), which showed a 69.17% decrease in TNF-α and an 82.11% decrease in IL-6 under the study conditions (Table 3).

### 3.7. Lung Tissue MMP-9 and MMP-12 Content

Significant increases in MMP-9 and MMP-12 levels were observed in the lung tissue of PM_2.5_ control mice compared to intact vehicle controls. The oral administration of AJ at all three doses significantly inhibited these PM_2.5_-induced elevations in a dose-dependent manner. Specifically, AJ at 400 mg/kg reduced MMP-9 levels by 57.99% and MMP-12 levels by 57.00% compared to PM_2.5_ controls. While effective, these reductions were less pronounced than those achieved with DEXA (0.75 mg/kg), which decreased MMP-9 by 68.98% and MMP-12 by 68.58% under the study conditions (Figure 4B).

### 3.8. Lung Tissue Levels of ACh and Substance P

Significant increases in lung tissue levels of acetylcholine (ACh) and substance P were observed in PM_2.5_ control mice compared to intact vehicle controls. These elevations were significantly inhibited by DEXA at 0.75 mg/kg. In contrast, AJ treatment at doses of 400, 200, and 100 mg/kg resulted in significant and dose-dependent increases in ACh and substance P levels. Specifically, substance P increased by 99.15% with AJ 400 mg/kg, while DEXA reduced it by 27.88%. Similarly, ACh increased by 97.44% with AJ 400 mg/kg, whereas DEXA decreased it by 26.97% compared to PM_2.5_ controls (Figure 4C).

### 3.9. Effects on Lung Lipid Peroxidation and Antioxidant Defense Systems

Significant increases in lung lipid peroxidation (elevated MDA content and ROS levels) and the depletion of antioxidant defenses (reduced GSH content, CAT, and SOD activities) were observed in PM_2.5_ control mice compared to intact vehicle controls. AJ administration (400–100 mg/kg) significantly mitigated these PM_2.5_-induced changes in a dose-dependent manner. Specifically, AJ at 400 mg/kg reduced pulmonary MDA by 41.36% and ROS levels by 50.18%. It also increased GSH content by 129.40%, SOD activity by 122.08%, and CAT activity by 184.85%. Although effective, these antioxidant effects were somewhat less pronounced than those observed with DEXA at 0.75 mg/kg, which showed stronger improvements across these parameters (Table 4).

### 3.10. mRNA Expression of Lung Tissue Genes Involved in Mucus Production

Significant increases in the mRNA expression of mucus production-related genes *MUC5AC* and *MUC5B* were observed in PM_2.5_ control mice compared to intact vehicle controls. AJ administration (400–100 mg/kg) significantly reduced these PM_2.5_-induced gene expression elevations in a dose-dependent manner. Notably, AJ at 400 mg/kg decreased *MUC5AC* expression by 47.83% and *MUC5B* expression by 34.36%, compared to PM_2.5_ controls. Although effective, these reductions were less pronounced than those achieved with DEXA at 0.75 mg/kg, which showed greater decreases in both *MUC5AC* and *MUC5B* expressions (Table 5).

### 3.11. mRNA Expression of Lung Tissue Genes Involved in Oxidative Stress and Inflammatory Processes: p38 MAPK, NF-κB, PI3K, PTEN, and Akt

In the PM_2.5_ control mice, there were substantial increases in mRNA expression levels of genes associated with oxidative stress and inflammation, including p*38 MAPKα*, *NF-κB1*, *Akt1*, *and PI3K*, along with a decrease in *PTEN* expression compared to intact vehicle controls. AJ administration at doses of 400, 200, and 100 mg/kg effectively inhibited these PM_2.5_-induced genetic changes in a dose-dependent manner. Specifically, AJ at 400 mg/kg reduced *NF-κB1* expression by 52.72%, *p38 MAPKα* by 54.84%, and *PI3K* by 59.71%. It also increased *PTEN* expression by 95.16%. These findings suggest that AJ has a protective effect against PM_2.5_-induced oxidative and inflammatory responses in lung tissue. However, the effects of AJ were somewhat less pronounced compared to DEXA at 0.75 mg/kg, which showed a 73.65% reduction in *NF-κB1* and a 67.20% reduction in *PI3K*, as well as a 113.23% increase in *PTEN* expression, demonstrating its potent modulatory capacity under the conditions of this study (Table 5).

### 3.12. mRNA Expression of Lung Tissue Genes Involved in Cell Apoptosis: Bcl-2 and Bax

In PM_2.5_ control mice, there were significant alterations in the mRNA expression of apoptosis-related genes: a decrease in the anti-apoptotic protein *Bcl-2* and an increase in the pro-apoptotic protein *Bax* compared to intact vehicle controls. AJ administration (400–100 mg/kg) significantly counteracted these PM_2.5_-induced changes in a dose-dependent manner. Specifically, AJ at 400 mg/kg increased *Bcl-2* expression by 81.84% and reduced *Bax* expression by 53.26% compared to PM_2.5_ controls. This suggests that AJ effectively mitigates PM_2.5_-induced pro-apoptotic signaling in lung tissue. However, the modulatory effects of AJ were less pronounced than those of DEXA at 0.75 mg/kg, which increased *Bcl-2* expression by 102.88% and reduced *Bax* expression by 62.39%, indicating a stronger capacity to restore the balance between pro- and anti-apoptotic signals under the study conditions (Table 5).

### 3.13. Lung Histopathological Observations

**Figure 5:**
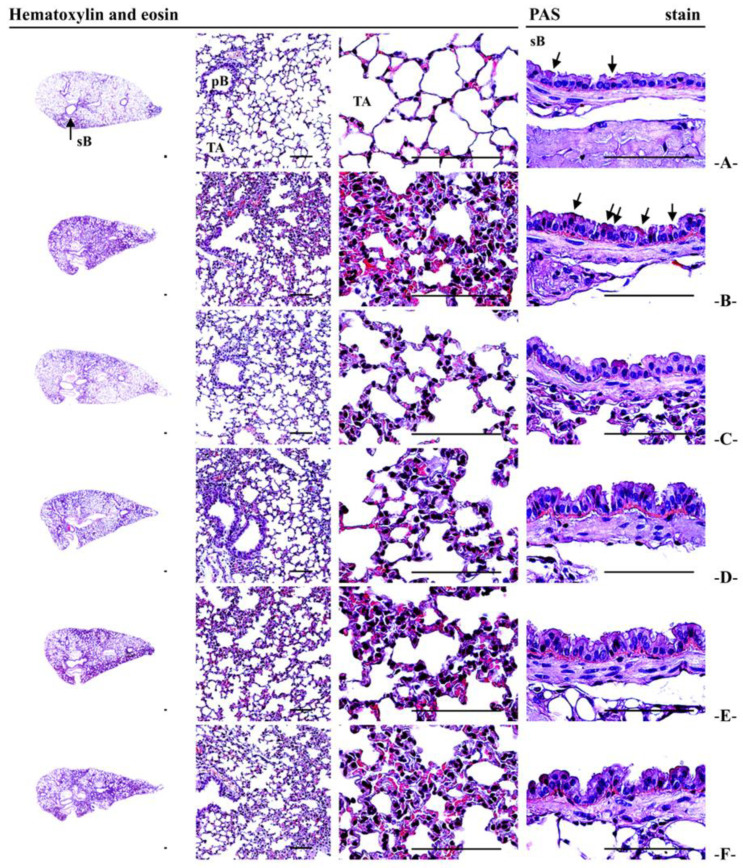
Representative histopathological profiles of lung left lobe tissues in mice with intact or PM_2.5_-induced pulmonary injury. (**A**) Intact vehicle control (mice orally administered with distilled water and given saline intranasal instillation), (**B**) PM_2.5_ control (mice orally administered with distilled water and given PM_2.5_ intranasal instillation), (**C**) DEXA (mice orally administered with 0.75 mg/kg DEXA and given PM_2.5_ intranasal instillation), (**D**) AJ_400_ (mice orally administered with 400 mg/kg AJ and given PM_2.5_ intranasal instillation), (**E**) AJ_200_ (mice orally administered with 200 mg/kg AJ and given PM_2.5_ intranasal instillation), (**F**) AJ_100_ (mice orally administered with 100 mg/kg AJ and given PM_2.5_ intranasal instillation). PM_2.5_ refers to diesel particulate matter NIST 1650b; DEXA denotes dexamethasone; AJ stands for Atractylodis Rhizoma, the root extract of *Atractylodes japonica* Koidz. ex Kitam; PAS represents Periodic Acid–Schiff; sB is secondary bronchus; pB denotes Primary Bronchiole; TA refers to Terminal Respiratory Bronchiole–Alveoli. Arrows point to PAS+ mucus-producing cells. Scale bars = 200 μm.

Following PM_2.5_ exposure, pronounced sarcomatous changes were observed, including inflammatory cell infiltration, the thickening of the alveolar septum, and the hyperplasia of PAS+ mucus-producing cells, confirmed through histopathological examination. In the PM_2.5_ control group, there were notable increases in the thickness of the secondary bronchus mucosa and alveolar septum and a reduction in airway surface area (ASA). AJ administration (400–100 mg/kg) significantly improved these histopathological changes in a dose-dependent manner. AJ at 400 mg/kg notably reduced alveolar septal thickness by 66.99%, and inflammatory cell infiltration by 47.26%, and increased ASA by 80.17% compared to PM_2.5_ controls. While effective, these improvements were less pronounced than those observed with DEXA at 0.75 mg/kg, which showed a 70.20% reduction in alveolar septal thickness and a 55.28% decrease in inflammatory cell infiltration. Additionally, AJ demonstrated strong expectorant activity by significantly increasing PAS+ mucus-producing cells and mucosa thickness in the secondary bronchus, with AJ at 400 mg/kg increasing PAS+ cells by 116.67% and mucosa thickness by 66.15% compared to PM_2.5_ controls, whereas DEXA did not significantly affect these parameters (Figure 5 and Table 6).

## 4. Discussion

Significant decreases in body weight were observed four days after administering DEXA at 0.75 mg/kg, compared to intact vehicle and PM_2.5_ control mice, aligning with previous studies indicating DEXA’s catabolic effects can induce weight loss [41]. In contrast, Atractylodis Rhizoma, the root of *A. japonica*, extracts (AJ extracts) at doses of 400 to 100 mg/kg did not affect body weight or weight gain, suggesting it does not interfere with normal growth. This characteristic is desirable for therapeutic agents targeting pulmonary injury without causing systemic side effects, which is consistent with findings from [42], who reported similar non-significant effects on body weight in analogous experimental setups. Moreover, the lung weight index, a marker of vascular permeability and edema, was significantly increased in PM_2.5_ control mice, indicating pulmonary congestion and edema, as seen in previous PM-induced lung injury models [2,3]. AJ administration significantly reduced these indices in a dose-dependent manner, although less effectively than DEXA, suggesting that AJ possesses anti-edematous properties. This corroborates findings by Tumes et al. [43] and Min et al. [20], who reported similar anti-inflammatory effects of AJ in respiratory models.

The liver function markers AST and ALT remained within normal ranges across all groups, indicating that neither PM_2.5_ exposure nor AJ and DEXA treatments induced hepatotoxicity. This supports the hepatic safety profile of AJ, corroborating studies such as Sodikoff [44], which highlight its potential as a safe therapeutic agent. The absence of significant changes in liver enzymes suggests that AJ can be safely administered without compromising liver function, even in the context of inflammatory challenges like PM_2.5_ exposure. Furthermore, intranasal PM_2.5_ instillation led to increased BALF cell counts, indicating inflammation, which AJ effectively reduced. This supports its role in modulating inflammatory responses, aligning with the anti-inflammatory activities reported in previous studies [19]. Although AJ’s reduction in inflammatory markers is less potent than DEXA, it points to its potential as a safer, albeit slightly less powerful, alternative. AJ’s ability to mitigate increases in total leukocytes, lymphocytes, neutrophils, eosinophils, and monocytes highlights its comprehensive anti-inflammatory potential.

Airway secretions are primarily regulated by acetylcholine (ACh) and substance P, both of which are known to increase with PM_2.5_ exposure, contributing to neurogenic inflammation and altered vascular responses [45,46,47]. These neurotransmitters control via substance P and ACh play a critical role as serous secretagogues, particularly affecting goblet and acinar cells [48]. Studies have consistently reported that PM_2.5_ exposure leads to elevated levels of ACh and substance P, which are associated with increased mucin production, specifically *MUC5AC* and *MUC5B* [47,48,49]. However, following a 10-day continuous oral administration of AJ, there were significant and dose-dependent reductions in the mRNA expressions of *MUC5AC* and *MUC5B*, indicating potent mucolytic expectorant effects. The increase in the levels of substance P and ACh following AJ treatment may be attributed to the compound’s ability to enhance serous secretions in the respiratory system. AJ appears to stimulate the neural pathways that regulate these neurotransmitters, promoting their release. This enhanced secretion acts as a compensatory mechanism to counteract the mucus overproduction induced by PM_2.5_ exposure. By increasing the levels of these neurotransmitters, AJ may facilitate the dilution and clearance of mucus, thereby exerting its mucolytic expectorant effects. This mechanism could be a response to the need for increased serous secretions to manage mucus hypersecretion disorders, as indicated by the observed dose-dependent reduction in mucin gene expressions (*MUC5AC* and *MUC5B*). These findings are supported by Na et al. [48] and Wang et al. [49], who observed similar effects in models of mucus overproduction. These findings underline the AJ’s potential in managing mucus hypersecretion disorders, such as chronic obstructive pulmonary disease (COPD) and asthma, by influencing the levels of key neurotransmitters involved in airway secretions.

The inhalation of PM elevated oxidative stress in the epithelial cells of the respiratory tract, leading to injury and inflammation [5,50]. Lipid peroxidation results in oxidative damage to cellular membranes, producing toxic aldehydes like malondialdehyde (MDA) [51,52]. Antioxidants such as GSH, SOD, and CAT help to mitigate this stress by reducing ROS levels, but decreased enzyme activity can signal an inability to counteract oxidative stress from PM_2.5_ exposure [1,2,3,53,54]. Similar studies have indicated that PM_2.5_ causes oxidative stress and inflammation, marked by increased ROS, MDA, and pro-inflammatory cytokines, including TNF-α and IL-6 [1,2,3,54]. AJ’s antioxidant properties help to attenuate these changes, though less effectively than DEXA [1,2,3]. NF-κB1 is crucial in ROS-induced inflammation [31,55], while PI3K/Akt and p38 MAPKα pathways are linked to oxidative stress and cancer pathogenesis, with PTEN levels decreasing in PM_2.5_-exposed mice [56,57]. PM_2.5_ exposure increased lung lipid peroxidation, ROS levels, and inflammatory markers while depleting antioxidants, but these effects were dose-dependently inhibited by AJ administration for 10 days. AJ reduced oxidative stress and inflammation by downregulating p38 MAPKα, PI3K/Akt, and NF-κB1 pathways. All AJ dosages demonstrated protective effects against PM_2.5_-induced pulmonary injuries, although their efficacy was slightly less than DEXA. The modulation of pathways by AJ provides insights into its mechanism of action, contributing to its protective effects against oxidative stress-induced pulmonary injury, which is significant given the role of oxidative stress in exacerbating respiratory conditions [31,58].

Matrix metalloproteinases (MMPs) play a crucial role in degrading extracellular matrix components and are involved in tissue remodeling and various disease processes [59]. Exposure to PM_2.5_ has been associated with the upregulation of MMP-9 and MMP-12 in the airways, contributing to airflow limitation and tissue destruction [60,61]. Oral AJ treatment at doses from 400 to 100 mg/kg significantly reduced the PM_2.5_-induced elevations in MMP-9 and MMP-12 in a dose-dependent manner, although it was not as effective as DEXA at 0.75 mg/kg, suggesting AJ’s protective effects against airway damage. Additionally, mitochondria, which are targeted by ROS, can suffer membrane damage and apoptosis through the mitochondrial pathway when exposed to excessive ROS [62,63]. PM_2.5_ exposure has been observed to decrease the expression of the anti-apoptotic protein *Bcl-2* and increase the expression of the pro-apoptotic protein *Bax*, leading to apoptosis [63,64]. This study demonstrated that AJ treatment at the same doses also significantly inhibited these PM_2.5_-induced apoptotic changes in a dose-dependent manner, providing protective effects against apoptosis.

The histomorphometrical index ASA (%/mm^2^) is crucial for assessing pulmonary functions like gas exchange capacities, where reductions indicate decreased lung capacity typical of various lung diseases [20,22,35]. PAS staining helps to identify mucus-producing cells, with increased intensity indicating higher cellular activity [34,65]. In addition, increases in bronchus mucosa thickness and PAS+ mucus-producing cells in the bronchus mucosa have been linked with expectorant effects [34,66]. PM_2.5_ exposure leads to pathological changes in mice, including inflammatory cell infiltration and alveolar septum thickening [2,3,19]. This study observed significant increases in alveolar septal thickness, bronchus mucosa thickness, inflammatory cell infiltration, and the number of PAS+ mucus-producing cells, along with reduced ASA in PM_2.5_-exposed mice. However, oral AJ treatment from 400 to 100 mg/kg significantly enhanced ASA and reduced septal thickness and inflammation in a dose-dependent manner, indicating potent expectorant effects [34,66]. DEXA did not significantly affect these parameters. These findings suggest that AJ, due to its expectorant, anti-inflammatory, and antioxidant properties, could be developed as an alternative respiratory treatment or functional food ingredient.

This study highlights the promising pulmonary protective effects of AJ, attributed to its anti-inflammatory, antioxidant, and mucolytic properties, alongside a favorable safety profile that supports its potential as a therapeutic agent. AJ emerges as a viable candidate for respiratory health development, either as a natural remedy or an adjunct to conventional treatments, despite DEXA’s greater potency. However, caution is advised in extrapolating these findings to clinical settings due to the absence of clinical data. Future research should delve into AJ’s long-term effects, underlying mechanisms, and potential synergistic interactions with existing therapies. The study’s findings suggest that the dose-dependent protective effects of AJ may be linked to its bioactive compounds, atractylenolide I and III, known for their expectorant, antioxidant, anti-inflammatory, and gastroprotective properties [67,68,69,70,71]. Despite these encouraging results, it remains uncertain whether the protective effects observed at a 400 mg/kg dosage represent a threshold or are influenced by bioavailability. Future studies should include detailed mechanistic investigations of protein content and phosphorylation changes, toxicological assessments, and evaluations across a broader range of dosages and efficacy in long-term PM_2.5_ exposure or alternative animal models, such as asthma. Moreover, while Atractylodis Rhizoma is recognized as a food item by the Korea Food and Drug Administration [72], the lack of data on potential toxicity and effects on hepatic metabolic enzymes and the lungs is a significant limitation. Additionally, acknowledging that changes in mRNA expression do not always correlate with protein levels via Western blot analysis, future investigations should incorporate direct assessments of protein levels to provide a more comprehensive understanding of AJ’s protective mechanisms.

## 5. Conclusions

This study investigated the lung-protective properties of Atractylodis Rhizoma (the root of *A. japonica* Koidz. ex Kitam) extracts (AJ extracts) on subacute pulmonary injury induced by PM_2.5_ in Balb/c mice. The oral administration of AJ from 400 to 100 mg/kg demonstrated significant protective effects by downregulating p38 MAPKα and PI3K/Akt pathways, thereby reducing oxidative stress, inflammation, and mucus production. AJ also exhibited antioxidant and anti-inflammatory activities, enhanced mucolytic expectorant effects, and increased lung substance P and ACh levels, without causing significant hepatotoxicity. While these effects were dose-dependent and less pronounced than those of DEXA (0.75 mg/kg), AJ uniquely showed mucolytic expectorant activities not observed in DEXA-treated mice. PM_2.5_ exposure resulted in significant lung damage, evidenced by increased inflammatory markers, oxidative stress, and histopathological changes, which were mitigated by 10 days of AJ administration. AJ treatment also led to decreased mRNA expressions related to mucus production and inflammation while improving mucus-related histopathological parameters. Consequently, AJ showed promise as a candidate for developing alternative respiratory therapies or functional food ingredients. While its lung-protective effects against injuries induced by PM_2.5_ are noteworthy, they are somewhat less effective than DEXA in certain areas. This warrants further research to explore AJ’s potential as a natural therapeutic agent for respiratory health.

## Figures and Tables

**Table 1:** Lung weights and gross observations in mice with intact or PM_2.5_-induced pulmonary injury.

Groups	Lung Weights		Congestional Regions (%)—Gross Findings
	Absolute (g)	Relative (%)	
Controls			
Intact vehicle	0.122 ± 0.005	0.627 ± 0.030	2.41 ± 1.68
PM_2.5_	0.179 ± 0.006 ^a^	0.908 ± 0.050 ^c^	62.02 ± 10.13 ^c^
Reference			
DEXA	0.123 ± 0.007 ^b^	0.706 ± 0.020 ^cd^	8.52 ± 3.28 ^cd^
Test substance—AJ			
400 mg/kg	0.144 ± 0.010 ^ab^	0.727 ± 0.032 ^cd^	14.68 ± 3.75 ^cd^
200 mg/kg	0.152 ± 0.007 ^ab^	0.774 ± 0.049 ^cd^	24.79 ± 10.55 ^cd^
100 mg/kg	0.158 ± 0.006 ^ab^	0.797 ± 0.054 ^cd^	37.11 ± 10.26 ^cd^

Values are presented as means ± SD for groups of 10 mice each. PM_2.5_ refers to diesel particulate matter NIST 1650b; DEXA stands for dexamethasone; AJ represents Atractylodis Rhizoma, the root extract of *Atractylodes japonica* Koidz. ex Kitam; THSD is Tukey’s Honest Significant Difference; DT3 denotes Dunnett’s T3. Significance is indicated as ^a^ *p* < 0.01 when compared with the intact vehicle control using the THSD test; ^b^ *p* < 0.01 when compared with the PM_2.5_ control using the THSD test; ^c^ *p* < 0.01 when compared with the intact vehicle control using the DT3 test; ^d^ *p* < 0.01 when compared with the PM_2.5_ control using the DT3 test.

**Table 2:** Cytological analysis of BALF in mice with intact or PM_2.5_-induced pulmonary injury.

Groups	Total Cells	Total Leukocytes	Differential Counts			
			Lymphocytes	Neutrophils	Eosinophils	Monocytes
Controls						
Intact vehicle	10.10 ± 2.56	6.60 ± 1.35	3.90 ± 1.20	1.05 ± 0.35	0.02 ± 0.02	1.12 ± 0.53
PM_2.5_	94.80 ± 14.90 ^c^	61.10 ± 11.84 ^c^	39.50 ± 11.68 ^c^	12.05 ± 1.57 ^a^	1.50 ± 0.28 ^c^	6.57 ± 1.16 ^c^
Reference						
DEXA	19.80 ± 3.16 ^ce^	11.90 ± 1.66 ^ce^	7.00 ± 1.25 ^ce^	2.26 ± 0.88 ^b^	0.06 ± 0.03 ^de^	1.85 ± 0.66 ^e^
Test substance—AJ						
400 mg/kg	41.30 ± 10.24 ^ce^	24.30 ± 4.90 ^ce^	15.10 ± 4.36 ^ce^	4.86 ± 0.88 ^ab^	0.30 ± 0.21 ^ce^	2.69 ± 0.80 ^ce^
200 mg/kg	58.40 ± 7.82 ^ce^	32.00 ± 4.83 ^ce^	20.10 ± 4.68 ^ce^	6.53 ± 1.13 ^ab^	0.53 ± 0.23 ^ce^	3.63 ± 0.63 ^ce^
100 mg/kg	66.50 ± 8.81 ^ce^	41.50 ± 4.72 ^ce^	27.30 ± 4.40 ^c^	7.60 ± 1.05 ^ab^	0.81 ± 0.17 ^ce^	4.39 ± 0.42 ^ce^

Values are presented as means ± SD for groups of 10 mice, with cell numbers expressed in ×10^4^ cells/mL. PM_2.5_ refers to diesel particulate matter NIST 1650b; DEXA stands for dexamethasone; AJ indicates Atractylodis Rhizoma, the root extract of *Atractylodes japonica* Koidz. ex Kitam; BALF denotes bronchoalveolar lavage fluid; THSD is Tukey’s Honest Significant Difference; DT3 represents Dunnett’s T3. Significance is marked as ^a^ *p* < 0.01 when compared with the intact vehicle control using the THSD test; ^b^ *p* < 0.01 when compared with the PM_2.5_ control using the THSD test; ^c^ *p* < 0.01 and ^d^ *p* < 0.05 when compared with the intact vehicle control using the DT3 test; ^e^ *p* < 0.01 when compared with the PM_2.5_ control using the DT3 test.

**Table 3:** Lung cytokine levels: *IL-6*, *TNF-α*, *CXCL1*, *and CXCL2* in mice with intact or PM_2.5_-induced pulmonary injury.

Groups	Lung Contents (pg/mL)			
	TNF-α	IL-6	CXCL1	CXCL2
Controls				
Intact vehicle	30.03 ± 10.58	30.70 ± 11.43	37.06 ± 11.37	17.09 ± 3.88
PM_2.5_	227.93 ± 65.55 ^c^	412.00 ± 56.09 ^c^	375.20 ± 117.27 ^c^	190.09 ± 24.98 ^a^
Reference				
DEXA	70.28 ± 12.42 ^cd^	73.70 ± 18.55 ^cd^	114.97 ± 28.13 ^cd^	58.79 ± 18.94 ^ab^
Test substance—AJ				
400 mg/kg	89.84 ± 16.89 ^cd^	137.64 ± 31.04 ^cd^	152.93 ± 36.92 ^cd^	76.21 ± 16.46 ^ab^
200 mg/kg	118.62 ± 22.21 ^cd^	206.01 ± 60.02 ^cd^	184.85 ± 22.35 ^cd^	102.45 ± 17.37 ^ab^
100 mg/kg	144.26 ± 15.80 ^ce^	266.09 ± 59.85 ^cd^	224.65 ± 21.31 ^ce^	129.45 ± 23.04 ^ab^

Values are presented as means ± SD for groups of 10 mice. PM_2.5_ refers to diesel particulate matter NIST 1650b; DEXA denotes dexamethasone; AJ stands for Atractylodis Rhizoma, the root extract of *Atractylodes japonica* Koidz. ex Kitam; TNF represents Tumor Necrosis Factor; IL stands for interleukin; CXCL refers to the chemokine (C-X-C motif) ligand; THSD is Tukey’s Honest Significant Difference; DT3 indicates Dunnett’s T3. Significance is noted as ^a^ *p* < 0.01 when compared with the intact vehicle control using the THSD test; ^b^ *p* < 0.01 when compared with the PM_2.5_ control using the THSD test; ^c^ *p* < 0.01 when compared with the intact vehicle control using the DT3 test; ^d^ *p* < 0.01 and ^e^ *p* < 0.05 when compared with the PM_2.5_ control using the DT3 test.

**Table 4:** Lung lipid peroxidation (MDA contents), GSH contents, and SOD and CAT activities in mice with intact or PM_2.5_-induced pulmonary injury.

Groups	Lung Contents (nM/mg Protein)			Lung Enzyme Activity (U/mg Protein)	
	MDA	ROS	GSH	SOD	CAT
Controls					
Intact vehicle	4.15 ± 1.14	27.23 ± 10.36	48.74 ± 13.90	331.90 ± 56.94	78.20 ± 16.16
PM_2.5_	20.55 ± 4.14 ^a^	90.03 ± 11.76 ^a^	6.39 ± 1.03 ^d^	72.00 ± 17.37 ^d^	9.90 ± 1.79 ^d^
Reference					
DEXA	6.36 ± 1.86 ^c^	44.05 ± 13.75 ^bc^	17.64 ± 4.24 ^de^	192.30 ± 45.78 ^de^	35.80 ± 11.51 ^de^
Test substance—AJ					
400 mg/kg	10.24 ± 1.24 ^ac^	52.79 ± 11.47 ^ac^	14.65 ± 3.44 ^de^	159.90 ± 23.25 ^de^	28.20 ± 12.35 ^df^
200 mg/kg	12.57 ± 2.23 ^ac^	59.04 ± 10.50 ^ac^	12.01 ± 2.30 ^de^	138.10 ± 21.37 ^de^	23.70 ± 5.58 ^de^
100 mg/kg	15.51 ± 1.69 ^ac^	65.52 ± 11.17 ^ac^	9.96 ± 1.42 ^de^	110.80 ± 12.33 ^de^	17.60 ± 5.02 ^df^

Values are presented as means ± SD for groups of 10 mice. PM_2.5_ refers to diesel particulate matter NIST 1650b; DEXA denotes dexamethasone; AJ stands for Atractylodis Rhizoma, the root extract of *Atractylodes japonica* Koidz. ex Kitam; MDA represents malondialdehyde; ROS indicates Reactive Oxygen Species; GSH stands for glutathione; CAT denotes catalase; SOD is superoxide dismutase; THSD refers to Tukey’s Honest Significant Difference; DT3 is Dunnett’s T3. Statistical significance is indicated as ^a^ *p* < 0.01 and ^b^ *p* < 0.05 when compared with the intact vehicle control using the THSD test; ^c^ *p* < 0.01 when compared with the PM_2.5_ control using the THSD test; ^d^ *p* < 0.01 when compared with the intact vehicle control using the DT3 test; ^e^ *p* < 0.01 and ^f^ *p* < 0.05 when compared with the PM_2.5_ control using the DT3 test.

**Table 5:** Alteration in lung tissue mRNA expression in mice with intact or PM_2.5_-induced pulmonary injury.

Groups	Controls		Reference	Test Substance—AJ		
	Intact Vehicle	PM_2.5_	DEXA	400 mg/kg	200 mg/kg	100 mg/kg
*MUC5AC*	1.00 ± 0.06	4.80 ± 0.67 ^a^	2.12 ± 0.71 ^ab^	2.50 ± 0.50 ^ab^	2.95 ± 0.44 ^ab^	3.45 ± 0.69 ^ab^
*MUC5B*	1.00 ± 0.05	2.88 ± 0.25 ^a^	1.64 ± 0.25 ^ab^	1.89 ± 0.21 ^ab^	2.06 ± 0.22 ^ab^	2.26 ± 0.23 ^ab^
*NF-κB* *1*	1.00 ± 0.04	9.18 ± 1.04 ^a^	2.42 ± 0.79 ^ab^	4.34 ± 1.39 ^ab^	5.67 ± 0.95 ^ab^	6.93 ± 1.05 ^ab^
*p38 MAPKα*	1.00 ± 0.04	7.33 ± 0.93 ^a^	2.90 ± 0.72 ^ab^	3.31 ± 0.52 ^ab^	4.64 ± 0.69 ^ab^	5.36 ± 0.47 ^ab^
*PTEN*	1.00 ± 0.05	0.31 ± 0.10 ^a^	0.66 ± 0.15 ^ab^	0.61 ± 0.11 ^ab^	0.54 ± 0.07 ^ab^	0.49 ± 0.03 ^ab^
*PI3K*	1.00 ± 0.06	7.02 ± 1.00 ^a^	2.30 ± 0.53 ^ab^	2.83 ± 0.58 ^ab^	4.00 ± 0.77 ^ab^	5.11 ± 0.61 ^ab^
*Akt* *1*	1.00 ± 0.05	5.09 ± 1.14 ^a^	1.90 ± 0.36 ^ab^	2.27 ± 0.31 ^ab^	2.88 ± 0.46 ^ab^	3.26 ± 0.18 ^ab^
*Bcl-2*	1.00 ± 0.06	0.35 ± 0.07 ^a^	0.70 ± 0.12 ^ab^	0.63 ± 0.11 ^ab^	0.57 ± 0.11 ^ab^	0.49 ± 0.04 ^ab^
*Bax*	1.00 ± 0.05	6.63 ± 0.93 ^a^	2.49 ± 0.40 ^ab^	3.10 ± 0.68 ^ab^	4.08 ± 0.75 ^ab^	4.67 ± 0.80 ^ab^

Values are presented as mean ± SD for groups of 10 mice, expressed as relative expressions normalized to β-actin mRNA. PM_2.5_ refers to diesel particulate matter NIST 1650b; DEXA denotes dexamethasone; AJ indicates Atractylodis Rhizoma, the root extract of *Atractylodes japonica* Koidz. ex Kitam; NF-κB is Nuclear Factor kappa-light-chain-enhancer of activated B cells; MAPK represents Mitogen-Activated Protein Kinases; PTEN is Phosphatase and Tensin Homolog, PI3K stands for Phosphoinositide 3-Kinase; Akt is Protein Kinase B; Bcl-2 refers to B-cell Lymphoma 2; Bax is Bcl-2-associated X Protein; DT3 denotes Dunnett’s T3. Statistical significance is indicated as ^a^ *p* < 0.01 when compared with the intact vehicle control using the DT3 test; ^b^ *p* < 0.01 when compared with the PM_2.5_ control using the DT3 test.

**Table 6:** Histomorphometrical analysis of lung left lobe tissue in mice with intact or PM_2.5_-induced pulmonary injury.

Groups	Mean ASA (%/mm^2^)	Mean Alveolar Septal Thickness (μm)	Mean Thickness of SB (μm)	Mean IF Cell Numbers Infiltrated in AR (×10 cells/mm^2^)	PAS-Positive Cells on the SB (cells/mm^2^)
Controls					
Intact vehicle	84.85 ± 6.23	3.79 ± 0.69	13.46 ± 1.28	31.60 ± 10.49	13.00 ± 4.03
PM_2.5_	40.27 ± 9.52 ^a^	42.53 ± 4.37 ^a^	17.25 ± 1.26 ^a^	527.50 ± 105.72 ^a^	38.40 ± 6.17 ^a^
Reference					
DEXA	78.91 ± 3.83 ^c^	12.68 ± 2.85 ^ac^	17.03 ± 1.85 ^a^	235.90 ± 59.60 ^ac^	36.40 ± 5.48 ^a^
Test substance—AJ					
400 mg/kg	72.55 ± 8.71 ^bc^	14.04 ± 2.19 ^ac^	28.67 ± 6.71 ^ac^	278.20 ± 51.00 ^ac^	83.20 ± 16.34 ^ac^
200 mg/kg	62.39 ± 6.38 ^ac^	23.15 ± 4.53 ^ac^	25.67 ± 4.22 ^ac^	328.40 ± 42.90 ^ac^	71.00 ± 16.20 ^ac^
100 mg/kg	57.65 ± 4.01 ^ac^	27.20 ± 4.79 ^ac^	22.79 ± 1.96 ^ac^	387.20 ± 38.84 ^ac^	60.60 ± 14.36 ^ac^

Values are presented as means ± SD for groups of 10 mice. PM_2.5_ refers to diesel particulate matter NIST 1650b; DEXA denotes dexamethasone; AJ indicates Atractylodis Rhizoma, the root extract of *Atractylodes japonica* Koidz. ex Kitam; ASA stands for Alveolar Surface Area; AR refers to alveolar region; SB denotes secondary bronchus mucosa; IF indicates inflammatory; PAS is Periodic Acid–Schiff; DT3 represents Dunnett’s T3. Statistical significance is indicated as ^a^ *p* < 0.01 and ^b^ *p* < 0.05 when compared with the intact vehicle control using the DT3 test; ^c^ *p* < 0.01 when compared with the PM_2.5_ control using the DT3 test.

## Data Availability

The original contributions presented in this study are included in the article and Supplementary Materials. Further inquiries can be directed to the corresponding authors.

## Supplementary Materials

The following supporting information can be downloaded at https://www.mdpi.com/article/10.3390/antiox14020127/s1. Figure S1. Schematic diagram of the manufacturing process for *Atractylodes japonica* extract; Table S1. Oligonucleotides utilized for quantitative RT-PCR; Table S2. Body weight gain in mice with intact or PM_2.5_-induced pulmonary injury.
