# Supplementary material for: Protective Effects of Atractylodis Rhizoma Extracts on Lung Injury Induced by Particulate Matter 2.5 in Mice
Source: Antioxidants (Basel). 2025 Jan 23;14(2):127. doi: 10.3390/antiox14020127 (PMC11851628; doi:10.3390/antiox14020127)
Supplement: Supplementary file 1 [file antioxidants-14-00127-s001.zip › antioxidants-3361237-supplementary.pdf]

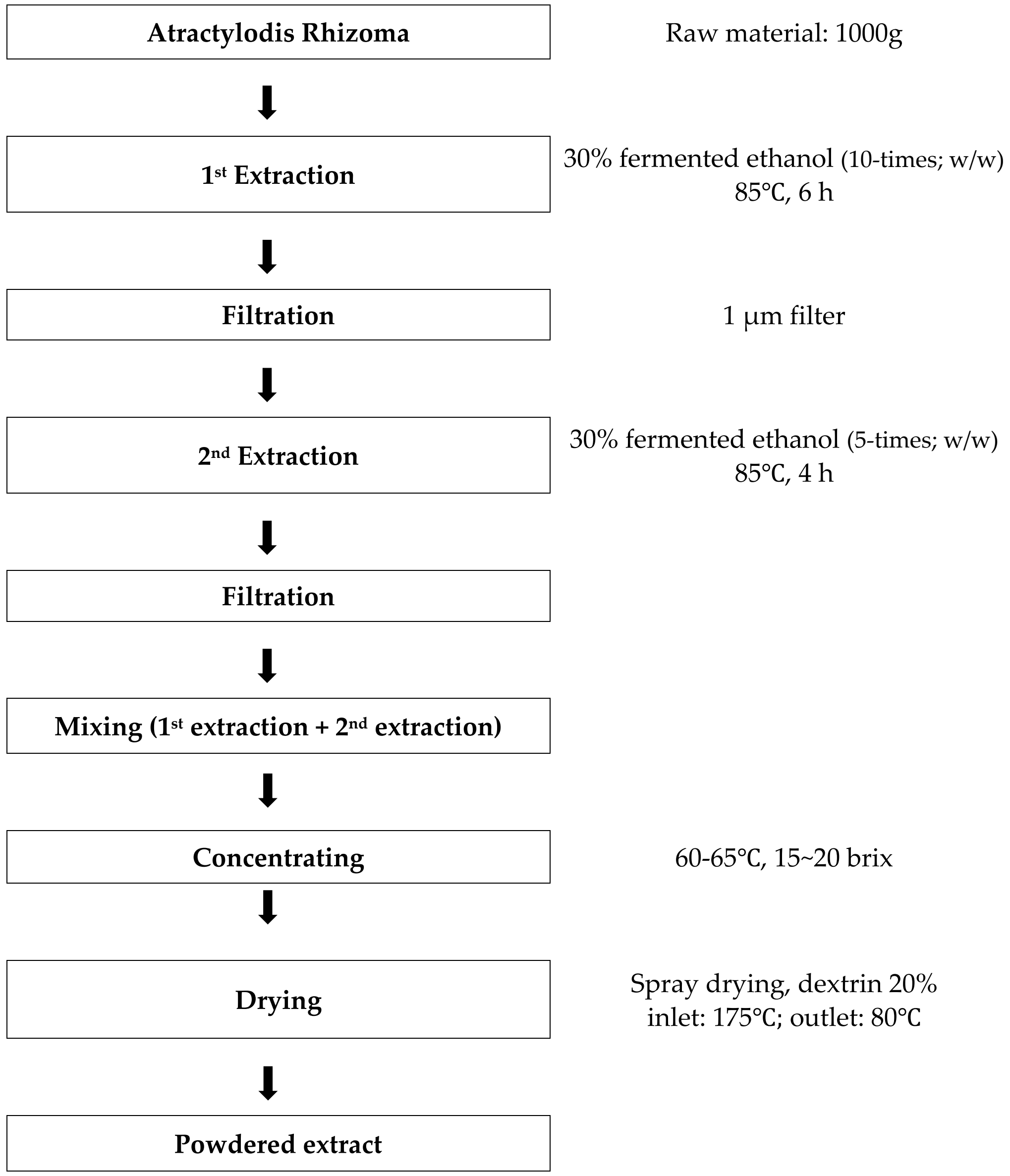

Figure S1. Schematic diagram of the manufacturing process for *Atractylodes japonica* extract.

Table S1. Oligonucleotides utilized for quantitative RT-PCR.

Target	5′ – 3′	Sequence	Gene ID
NFκB1	Forward<br>Reverse	CAATGGCTACACAGGACCA<br>CACTGTCACCTGGAACCAGA	18033
PTEN	Forward<br>Reverse	TGGATTCGACTTAGACTTGACCT<br>GCGGTGTCATAATGTCTCTCAG	19211
p38 MAPKα	Forward<br>Reverse	CGTTGTTTCCTGGTACAGACC<br>CCATTTCTTCTTGGTCAAGGG	26416
PI3K	Forward<br>Reverse	TCCAAATACCAGCAGGATCA<br>ATGCTTCGATAGCCGTTCTT	18708
Akt1	Forward<br>Reverse	TACTCATTCCAGACCCACGA<br>GAGGTTCTCCAGCTTCAGGT	11651
MUC5AC	Forward<br>Reverse	CACCATCTCTACAACCCAAACT<br>TGAGGTCCAGGTCTTTGTGTCT	17833
MUC5B	Forward<br>Reverse	GCCCTCACTGCCTCTGCTCCAC<br>TTTTACAGTGCCAGG GTTTATT	74180
Bcl-2	Forward<br>Reverse	ACCCTGTTGTGTAGCCGTCTG<br>GCATCCCAGCCTCCGTTATCA	12043
Bax	Forward<br>Reverse	GCTTACCGTAGCAGTTGGAT<br>GCCTTGAGCACCAGTTTGCT	12028
β-actin	Forward<br>Reverse	CTGTCGAGTCGCGTCCACCCGCGAG<br>CTCGCGGGTGGACGCGACTCGACAG	11461

RT-PCR: Reverse Transcription Polymerase Chain Reaction; NF-κB: Nuclear Factor kappa-light-chain-enhancer of activated B cells; MAPK: Mitogen-Activated Protein Kinases; PTEN: Phosphatase and Tensin Homolog; PI3K: Phosphoinositide 3-Kinase; Akt: Protein Kinase B; Bcl-2: B-Cell Lymphoma 2; Bax: Bcl-2-Associated X Protein.

Table S2. Body weight gain in mice with intact or $PM_{2.5}$-induced pulmonary injury.

Groups	Body weights at			Body weight gains [B-A]
	**Initial test article administration** [A]*	**Last test article administration**	**24 h after last test article administration** [B]*	
Controls				
Intact vehicle	18.18 ± 0.72	22.61 ± 0.84	19.42 ± 0.80	1.24 ± 0.50
$PM_{2.5}$	18.50 ± 0.88	22.70 ± 0.74	19.76 ± 0.79	1.26 ± 0.46
Reference				
DEXA	18.50 ± 0.96	19.76 ± 1.17[ab]	17.47 ± 1.03[ab]	- 1.03 ± 0.45[ab]
Test substance – AJ				
400 mg/kg	18.43 ± 1.21	22.66 ± 0.94	19.76 ± 0.82	1.33 ± 0.71
200 mg/kg	18.41 ± 1.45	22.68 ± 1.01	19.63 ± 1.06	1.22 ± 0.84
100 mg/kg	18.60 ± 1.03	22.93 ± 1.02	19.89 ± 1.07	1.29 ± 0.34

Values are presented as means ± SD for groups of 10 mice, measured in grams. $PM_{2.5}$ refers to diesel particulate matter NIST 1650b; DEXA denotes dexamethasone; AJ stands for Atractylodis Rhizoma, the root extract of *Atractylodes japonica* Koidz. ex Kitam; THSD represents Tukey's Honest Significant Difference test. All animals were fasted overnight for 18 hours, with water available. Statistical significance is indicated as [a] $p < 0.01$ when compared with the intact vehicle control using the THSD test; [b] $p < 0.01$ when compared with the $PM_{2.5}$ control using the THSD test.
